# Environmentally friendly and breathable wet-laid hydroentangled nonwovens for personal hygiene care with excellent water absorbency and flushability

**DOI:** 10.1098/rsos.171486

**Published:** 2018-04-18

**Authors:** Chao Deng, Wanjun Liu, Yinjiang Zhang, Chen Huang, Yi Zhao, Xiangyu Jin

**Affiliations:** Engineering Research Center of Technical Textiles, Ministry of Education, College of Textiles, Donghua University, No. 2999 North Renmin Road, Songjiang, Shanghai 201620, People's Republic of China

**Keywords:** hydroentanglement, wet-laid technique, nonwoven, pulp fibres, Tencel fibres

## Abstract

Developing wet-laid papers with a good wet strength remains a longstanding challenge in the papermaking industry. In this study, hydroentanglement, a mechanical bonding technique is developed to consolidate the wet-laid fibre web. The results indicate that wet tensile strength, ductile stretching property, softness, air permeability and water absorbency of the wet-laid fibre web are significantly improved by hydroentanglement. In addition, the abrasion test shows that the dusting off rate of wet-laid fibre web can be effectively reduced through hydroentanglement. Moreover, the disintegration experiment proves that wet-laid hydroentangled nonwovens could be easily dispersed when compared with conventional carded hydroentangled nonwovens. Therefore, the new wet-laid hydroentangled nonwovens can maintain excellent performance in a wet state, showing a great potential for personal hygiene applications.

## 1. Introduction

In recent years, the demand of wet-laid materials for personal hygiene care, beauty care, medical care, household wipe cleaning and industrial cleaning has grown significantly owing to the improvement of living standards [1–6]. Wet-laid materials refer to papers, wet napkin, nonwovens, etc. [7–10]. Among them, papers are mostly widely used owing to their good degradability and high water absorption property [11–13].

Papers are usually produced through a fibre web forming *via* wet-laid technique and subsequent bonding process [14–16]. The formation of fibre web involves the production of fibre slurry, where fibres with a length up to 35 mm [17] can be processed. Apart from wood pulp fibres, a range of fibres including polyester fibres, polyamide fibres, carbon fibres, ceramic fibres, glass fibres and cellulose fibres can also be manufactured through the wet-laid technique.

For the bonding process, a wet strength agent is an effective way to achieve inter-fibre bonding in wet-laid fibre web. Urea formaldehyde resins are commonly used to improve wet strength, but such wet strength is weak as the resins are difficult to combine with fibres to achieve efficient retention [18]. The wet strength has been improved by adopting melamine formaldehyde resins as the wet strength agent. However, these resins are harmful to both human body and environment [19–21]. Polyethyleneimine can only be used for absorbent wet paper without sizing treatment, and dialdehyde starch can only provide materials with temporary wet strength [22–24]. To overcome these limitations, a type of efficient wet strength agent, polyamide epichlorohydrin resin has been used to improve the wet strength without losing the softness and absorbency of wet-laid materials [25–27]. This wet strength agent may be a good choice for various applications such as personal hygiene care and medical care without regard to environmental pollution.

Hydroentanglement is widely used in the textile industry for fabricating nonwoven materials, which consolidate the fibre web by creating fibre entanglements and thus fibre-to-fibre friction force through fine high pressure water jets. During the hydroentanglement process, the high-speed water jets hit the delivery belt and form random reflection after passing through the fibre web. These direct and reflected water jets initiate intense agitations in the fibre web, leading to considerable fibre entanglements and improved mechanical property [28,29]. The hydroentanglement technique generates no pollution as most water can be recirculated and the technique does not involve any chemical additives.

It should be noted the fibre web is usually produced by carding, air-laid and wet-laid techniques in the nonwoven industry [30]. However, pulp fibres ranging from 2.4 to 2.6 mm in length are too short to process through conventional carding techniques which requires fibre with a length from 15 to 200 mm [30]. Although the pulp fibre length can meet the requirement of the air-laid technique, 4%–5% of the wood pulp fibres of the air-laid fibre web would be lost during the following hydroentanglement process if the fibre web is not pre-bonded [30].

In this paper, eco-friendly wet-laid and hydroentanglement techniques are combined to improve the wet strength of papers. Basic properties of nonwovens produced by wet-laid and hydroentanglement techniques are investigated. In addition, the softness, air permeability, water absorbency, dusting off properties and dispersibility of nonwovens are examined, which are important for practical applications.

## 2. Material and methods

### 2.1. Materials

Commercial pulp fibres and Tencel fibres are supplied by Canfor Corporation (Prince George, British Columbia, Canada) and Lenzing Fibers Co., Ltd (Nanjing, Jiangsu Province, China). The general properties of the fibres are summarized in table 1. Bathroom tissue is purchased from BREEZE^®^ (GOLD Hongye Paper Group Co., Ltd, Suzhou, China).

**Table 1.** General properties of the pulp and Tencel fibres.

properties	pulp fibres	Tencel fibres
fibre length (mm)	2.4–2.6	12
moisture content (%)	6.8	11.0
colour	white	white
water swelling	moderate	moderate

### 2.2. Fabrication of nonwovens by wet-laid and hydroentanglement techniques

#### 2.2.1. Fabrication of fibre web through wet-laid technique

**Figure 1.**
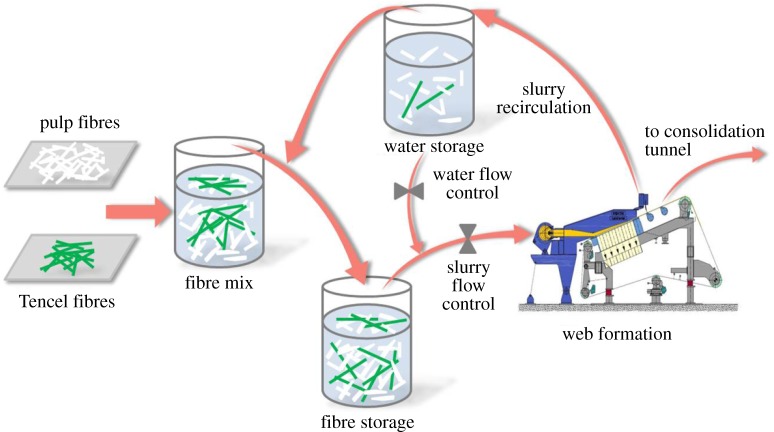
Schematic diagram of the wet-laid process with a slurry dispersion and a water recovery system.

The wet-laid process is shown in figure 1. Specifically, pulp and Tencel fibres with a weight ratio of 4 : 1 are weighed and mixed in a fibre mixing chest (60 m^3^). The concentration of fibres in the water dispersion is 15 g l^−1^. The mixing process is carried out for 20 min with a vigorous agitation of 1475 r min^−1^. Then the mixed fibre slurry is transferred to the fibre storage chest (30 m^3^), and sequentially diluted to 1 g l^−1^ and 0.4 g l^−1^ with a less vigorous agitation of 975 r min^−1^ (10 min each procedure). Lastly, the fibre slurry is pumped to the hydroformer (Voith Paper GmbH, Heidenheim, Germany) cover strip with a continuous speed of 150 m min^−1^. Then the fibre web (named as wet-laid fibre web for the following discussion) is formed on the cover strip by removing the water of fibre slurry *via* vacuum.

#### 2.2.2. Consolidation of wet-laid fibre web through hydroentanglement

**Figure 2.**
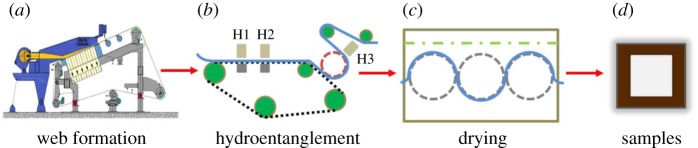
Schematic diagram for the fabrication of wet-laid hydroentangled nonwovens.

Here, hydroentanglement (Fleissner Aquajet 3-3600-8, Trützschler Nonwovens & Man-Made Fibers GmbH, Egelsbach, Germany) is carried out to consolidate the fibre web into nonwoven materials (figure 2). The hydroentanglement process involves a long belt with two rows of water jets (H1 and H2) and a subsequent drum with one row of water jets (H3). The main parameters of the hydroentanglement process are summarized in table 2. Finally, the nonwovens (named as wet-laid hydroentangled nonwovens) are dewatered by the suction device (Trützschler Nonwovens & Man-Made Fibers GmbH, Egelsbach, Germany), dried (Trützschler Nonwovens & Man-Made Fibers GmbH, Egelsbach, Germany) and rolled (A. Celli Nonwovens GmbH, Lucca, Italy).

**Table 2.** Parameters of the hydroentanglement process.

long belt hydroentanglement stage	pressure of the jet head 1 (bar)	30.00
	pressure of the jet head 2 (bar)	30.00
	the diameter of the water pinhole (mm)	0.12
	speed of the forming belt (m min^−1^)	150.00
drum hydroentanglement stage	pressure of the jet head 3 (bar)	35.00
	the diameter of the water pinhole (mm)	0.12
	speed of the forming belt (m min^-1^)	150.00

### 2.3. Characterization

#### 2.3.1. Morphology characterization by scanning electron microscope

The surface and cross-section of wet-laid fibre web and wet-laid hydroentangled nonwovens are observed by scanning electron microscope (SEM, TM3000, Hitachi, Tokyo, Japan) at a voltage of 15 kV. The samples are coated with a gold–platinum alloy in a sputter coater (LDM150D, Taiwan Jingda, China).

#### 2.3.2. Characterization of mechanical properties

The tensile strength, modulus and breaking elongation are examined using an electronic fabric strength tester (YG026MB, Wenzhou Fangyuan Instrument Co., Ltd, Wenzhou, China). Specifically, the moisture content of samples for the wet tensile test, the size of the samples for both longitudinal (MD) and transverse (CD) directions, the clamp distance, and the stretching rate are 200%, 50 × 250 mm^2^, 200 mm and 100 mm min^−1^, respectively.

#### 2.3.3. Evaluation of softness

The softness property of the wet-laid fibre web, wet-laid hydroentangled nonwovens and bathroom tissue are determined using a nonwoven & textile softness analyzer TSA (Emtec Electronic GmbH, Leipzig, Germany). Specifically, a sample in diameter of 112.8 mm is firstly fixed on a measuring cell, and then a mobile measuring blade with a body of rotation is pressed onto the sample with a defined force. The resulting vibrations of the measuring blade are detected with a sensor, converted into decibel values, and then decibel values of surface smoothness and surface softness are analysed at 500–550 Hz and 6600–6650 Hz range, respectively. The stiffness is represented by the displacement value measured under a fixed force on samples. In this method, three basic parameters including surface smoothness, surface softness, and stiffness are measured, which mainly influence the haptic characteristics [31], thus determining the softness properties.

#### 2.3.4. Measurement of air permeability, water absorption property and pore size distribution

The air permeability is evaluated using a digital air permeability tester (YG461E, Wenzhou Fangyuan Instrument Co., Ltd, Wenzhou, China). The dimension of the samples is 250 × 250 mm^2^.

To measure the water absorption rate, samples (100 × 100 mm^2^) with an original mass of *W*_*o*_ are placed in distilled water for 1 min, then the samples are taken out and hung vertically for 3 min, after that the mass of the samples are weighed as *W_a_*. The water absorption rate *W_m_* is calculated as follows:

2.1 $$W_{m}=\frac{W_{a}-W_{o}}{W_{o}}\times 100\;\%.$$

A capillary flow porometer (CFP-1100AI, Porous Materials Inc., USA) is used to investigate the pore size and distribution of the wet-laid fibre web, wet-laid hydroentangled nonwovens and bathroom tissue.

The temperature and relative humidity for these tests are kept constant at 20 ± 2°C and 65% ± 4%, respectively.

#### 2.3.5. Examination of dusting off

The dusting off property is evaluated using a fabric abrasion tester (YG401E, Ningbo Textile Instrument Factory, Ningbo, China). The diameter of the samples, pressure imposed on samples, temperature and relative humidity are $38.0_{0}^{+0.5}\;\text{mm}$, 12 kPa, 20 ± 2°C and 65% ± 4%, respectively.

#### 2.3.6. Detection of dispersibility

The dispersibility property is detected using a magnetic stirrer (RS-1DN, AS ONE Corporation, Osaka, Japan) according to the Guidelines for Assessing the Flushability of Disposable Nonwoven Products (third edition). Samples with an original mass of *M* are put into the beaker on the magnetic stirrer for disintegration. After 10 min, the contents of the beaker are rinsed through a 12.5 mm perforated plate sieve. The materials retained on the sieve are recovered and weighted as *M*_1_. Hence, the dispersion rate *R_d_* is calculated as follows:

2.2 $$R_{d}=\frac{M-M_{1}}{M}\times 100\;\%.$$

The size of the samples, liquid volume, rotational speed, temperature and relative humidity are 50 × 50 mm^2^, 600 ml, 400 r.p.m., 20 ± 2°C and 65% ± 4%, respectively.

#### 2.3.7. Statistical analysis

It should be mentioned that at least three samples are tested unless otherwise noted. The values are expressed as average ± s.d. The experimental data adopt one-way analysis of variance and the *α*-value is set at 0.05. If *F *> *F*-crit, the differences among data are considered statistically significant and marked by an asterisk (*).

## 3. Results and discussion

### 3.1. Parameters of raw materials

**Figure 3.**
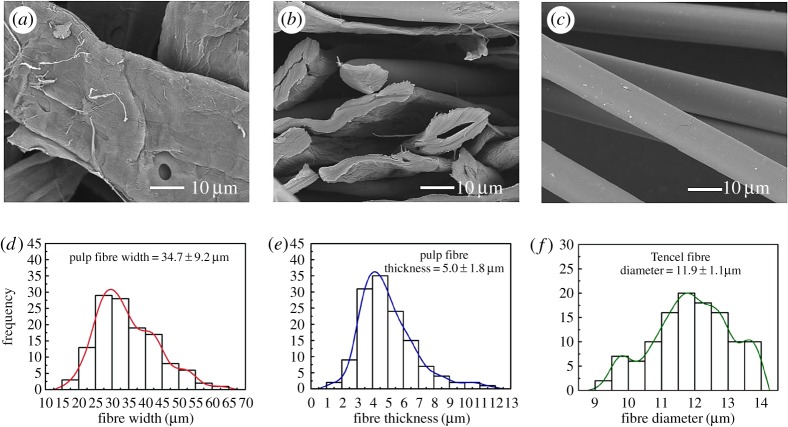
(*a–b*) Pulp fibres. (*c*) Tencel fibres. (*d*) The width distribution of pulp fibres. (e) The thickness distribution of pulp fibres. (*f*) The diameter distribution of Tencel fibres.

In this study, wet-laid and hydroentanglement techniques are employed for the fabrication of fibre web and fibre web consolidation, respectively (figure 2). Besides pulp fibres, Tencel fibres, a kind of cellulose fibres are used owing to their high wet strength and modulus. SEM images of pulp and Tencel fibres can be seen in figure 3*a–c*. It should be noted that the pulp fibres exhibit a ribbon structure with a width and a thickness of 34.7 ± 9.2 and 5.0 ± 1.8 µm, respectively (figure 3*d,e*), while the Tencel fibres have a circular cross-section in diameter of 11.9 ± 1.1 µm (figure 3*f*).

### 3.2. Characterization of wet-laid fibre web and wet-laid hydroentangled nonwovens

**Figure 4.**
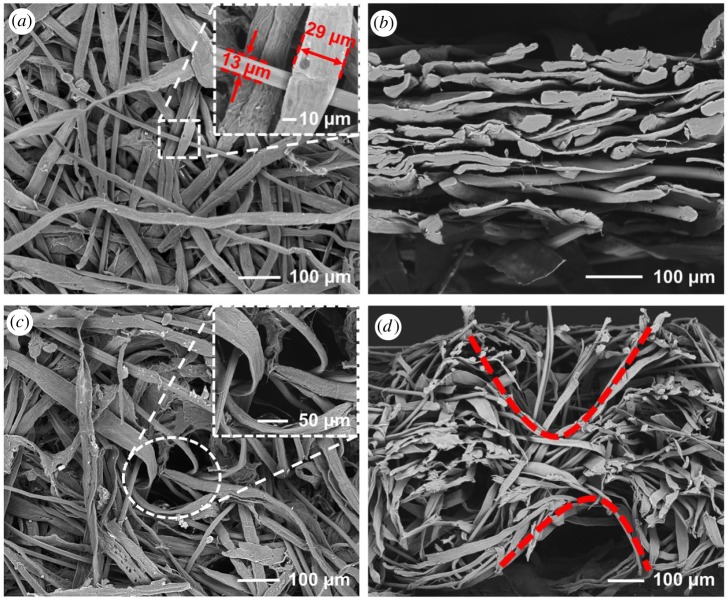
SEM images of wet-laid fibre web and wet-laid hydroentangled nonwovens showing the effect of hydroentanglement on the wet-laid fibre web. (*a*) Surface structure and (*b*) cross section of wet-laid fibre web. (*c*) Surface structure and (*d*) cross section of wet-laid hydroentangled nonwovens.

During the wet-laid process, pulp and Tencel fibres with a ratio of 4 : 1 (w/w) are used to produce the fibre web. For the fibre web produced by wet-laid technique, no obvious entangling structures between fibres are found. By contrast, most Tencel fibres and pulp fibres are stacked on each other tightly, orienting in the same plane as the sheet, within relatively narrow hypothetical layers (figure 4*a*,*b*). This phenomenon should be attributed to the combined principles of filtration and thickening during the wet-laid process. The water in the slurry is drained and the fibres are deposited on the forming wire randomly under the effect of suction.

After hydroentanglement, distinct entangling structures between pulp and Tencel fibres are found in the wet-laid hydroentangled nonwovens (figure 4*c*,*d*). During the hydroentanglement process, the high pressure water jets are applied on the wet-laid fibre web, making the surface pulp and Tencel fibres intersperse and move downwards, so these surface fibres are entangled with the fibres in the interior. We can also see that the cross-sectional structures of the wet-laid fibre web are changed after hydroentanglement (figure 4*d*). The trace of a water jet pinhole can be found in figure 4*d*. During the hydroentangling process, fibres on the surface are penetrated inside the fibre web materials, some of them form a V-type structure under the impact of water jets. Entanglements between fibres increase rather than stacking on each other. Fibres penetrated into the bottom surface are penetrated inside the material again under the impact of reflective water jets, forming more obvious entangling structures. It is also worth noting that the splitting of pulp fibres make materials become fluffy after consolidation, which is believed to have a positive effect on the softness, air permeability and water absorption properties of nonwovens.

### 3.3. Mechanical properties

**Figure 5.**
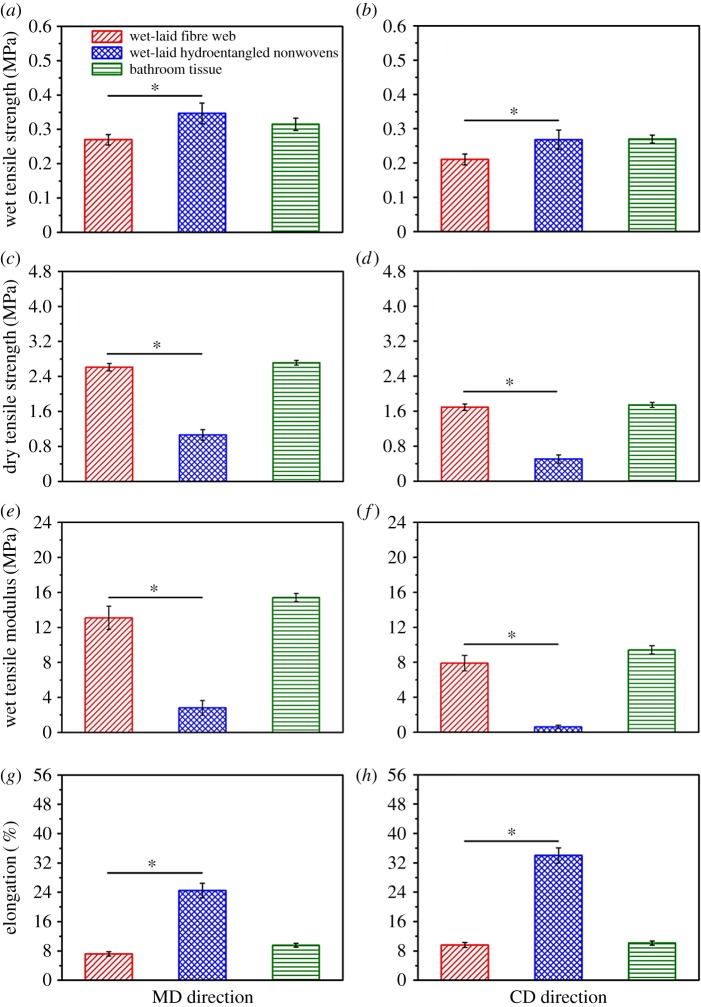
Comparison of tensile strength, modulus and elongation of wet-laid fibre web, wet-laid hydroentangled nonwovens and bathroom tissue at MD and CD directions. (*a–b*) Wet tensile strength at MD and CD directions. (*c–d*) Dry tensile strength at MD and CD directions. (*e–f*) Wet tensile modulus at MD and CD directions. (*g–h*) Elongation at MD and CD directions.

Mechanical strength is a very significant property for the application of wet-laid hydroentangled nonwovens, especially in a wet state. Comparison of wet tensile strength of wet-laid fibre web, wet-laid hydroentangled nonwovens and bathroom tissue at MD and CD directions are shown in figure 5*a*,*b*. The wet tensile strength values of these three kinds of materials at MD direction are 0.27 ± 0.02, 0.35 ± 0.03 and 0.32 ± 0.02 MPa, respectively (figure 5*a*). The tensile strength of the wet-laid fibre web in wet conditions is lower than that of wet-laid hydroentangled nonwovens owing to lack of fibre entanglements, confirmed by SEM images (figure 4*a*,*b*). For the wet-laid hydroentangled nonwovens, the existence of fibre entanglements between pulp and Tencel fibres significantly improves the mechanical properties in a wet state. These results indicate that hydroentanglement might be able to enhance the mechanical property of wet-laid fibre web in a wet condition. Moreover, the wet tensile strength of wet-laid hydroentangled nonwovens is comparable to that of bathroom tissue (figure 5*a*), demonstrating that the wet-laid hydroentangled nonwovens could have a potential application in personal hygiene care in terms of mechanical performance. In addition, wet tensile strength values of the wet-laid fibre web, wet-laid hydroentangled nonwovens and bathroom tissue in CD direction are 0.21 ± 0.02, 0.27 ± 0.03 and 0.27 ± 0.01 MPa, respectively (figure 5*b*). It should be noted that wet tensile strength of these three materials at MD direction is higher than that at CD direction as the intrinsic nature of the hydroformer station promotes fibre alignment in the MD (preferential) direction.

Regarding the dry tensile strength, interesting results can be found in figure 5*c*,*d*. The dry tensile strength values of the wet-laid fibre web, wet-laid hydroentangled nonwovens and bathroom tissue are 2.61 ± 0.09, 1.06 ± 0.12, 2.71 ± 0.05 MPa at MD direction, and 1.70 ± 0.07, 0.51 ± 0.09, 1.74 ± 0.06 MPa at CD direction, respectively. The dry tensile strength of the wet-laid fibre web is much higher than that of wet-laid hydroentangled nonwovens, which is opposite to the tendency of wet tensile strength. This phenomenon could be explained by the fact that wet-laid fibre web has a structure that fibres mostly stay in-plane, resulting in stronger hydrogen bonds and thus have higher tensile strength at dry status. While hydroentanglement makes the fibre orientate away from being mostly in-plane, it is logical that wet-laid hydroentangled nonwovens have weaker tensile strength at dry status. However, in wet status physical entanglements mainly contribute to the mechanical properties, endowing wet-laid hydroentangled nonwovens with better mechanical properties than that of wet-laid fibre web. With regard to bathroom tissue, the dry tensile strength of the wet-laid fibre web is lower than that of bathroom tissue, which should be attributed to the added binder in the bathroom tissue.

As for the wet tensile modulus, a huge decrease from 13.1 ± 1.3 to 2.8 ± 0.8 MPa of wet-laid fibre web is shown at MD direction after hydroentanglement (figure 5*e*). This phenomenon could be attributed to the resultant fluffy and bulkier structure induced by the hydroentanglement process, and thus out-of-plane orientation of fibres leading to lower within-plane strength. The wet tensile modulus of bathroom tissue is 15.4 ± 0.5 MPa, which is the highest among these three materials. This higher wet tensile modulus could result from the binder and wet strength agent added in the bathroom tissue during the fabrication process. The same relationship of these three materials at CD direction can be seen in figure 5*f*. The values of wet-laid fibre web, wet-laid hydroentangled nonwovens and bathroom tissue are 7.9 ± 0.9, 0.6 ± 0.2 and 9.4 ± 0.5 MPa, respectively, which are lower than those in MD direction owing to the fibre orientation distribution during the wet-laid process.

Besides tensile strength and modulus, the breaking elongation of these three materials is also investigated. Because nonwovens belong to textile materials, the ductile stretching property should also be considered. Comparing to wet-laid fibre web, the breaking elongations of wet-laid hydroentangled nonwovens in a wet state increase from 7.2% ± 0.6% to 24.5% ± 2.0% at MD direction, from 9.6% ± 0.6% to 34.0% ± 2.0% at CD direction, respectively (figure 5*g*,*h*). According to the SEM images (figure 4*a*,*b*), wet-laid fibre web has paper-like structures which are relatively condensed owing to the filtration and thickening processes during the wet-laid process, while wet-laid hydroentangled nonwovens have more ductile stretching property owing to the formation of fibre entangling and fluffy structures (figure 4*c,d*). The elongation values of bathroom tissue are 9.6% ± 0.5% and 10.1% ± 0.6% at MD and CD directions, respectively (figure 5*g*,*h*), which are lower than those of wet-laid hydroentangled nonwovens. These results indicate that the wet-laid hydroentangled nonwovens could provide better mechanical properties for use in personal hygiene care.

### 3.4. Softness

**Figure 6.**
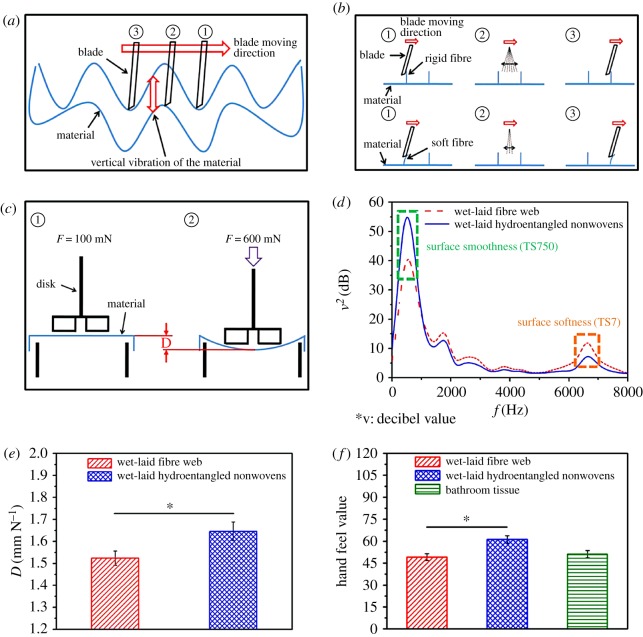
Softness improvement of wet-laid fibre web through hydroentanglement. Principle of the determination of (*a*) surface smoothness, (*b*) surface softness, and (*c*) displacement. (*d*) Determination of the surface smoothness and surface softness. (*e*) The displacement. (*f*) Hand feel value.

The application of wet-laid hydroentangled nonwovens in personal hygiene care requires good softness properties. For this reason, softness behaviours of wet-laid fibre web, wet-laid hydroentangled nonwovens and bathroom tissue are evaluated. There are three basic parameters: surface smoothness (TS750 values), surface softness (TS7 values) and stiffness (D values) of materials which determine the softness [31]. The surface smoothness of materials is tested through the vertical vibration of the materials caused by the horizontal motion of the blade on the surface structures (figure 6*a*). The surface softness of materials is tested through the vibration of the blade itself by moving it over the fibres on the surface of tested materials (figure 6*b*). These vibrations are detected by a vibration sensor and converted into the decibel values. Big vibrations result in high decibel values. The resulting displacement between a load of 100 and 600 mN of the material represents the stiffness, which correlates to the bulk softness (figure 6*c*). Hand feel (HF) value is a comprehensive value which combines these three main parameters and gives the specific softness evaluation of nonwoven materials. As can be seen in figure 6*d*, every sound spectrum curve has two evident peaks, the first peak represents the surface smoothness (TS750 values) of materials, and the second represents the surface softness (TS7 values). It can be seen that TS750 values of both wet-laid fibre web and wet-laid hydroentangled nonwovens are very high in the 0.2–2 kHz range. The decibel value of wet-laid fibre web (40.4 dB) is lower than that of wet-laid hydroentangled nonwovens (54.6 dB), indicating that wet-laid fibre web has a better surface smoothness, which is consistent with the microstructure in SEM images. In the high frequencies range (at approx. 6500 Hz), wet-laid fibre web has higher decibel value (11.7 dB) than that of wet-laid hydroentangled nonwovens (7.2 dB), which indicates that the surface materials become softer after hydroentanglement. This is owing to the fact that the water jets lead to the splitting of pulp fibres, which makes the fibres finer and softer. Besides the surface smoothness and surface softness, stiffness is another important parameter. In figure 6*e*, the *D* value of wet-laid fibre web (1.52 ± 0.03 mm N^−1^) is slightly lower than that of wet-laid hydroentangled nonwovens (1.65 ± 0.04 mm N^−1^). This could be attributed to the fluffy structure of wet-laid hydroentangled nonwovens induced by high pressure water jets. The comprehensive HF value of wet-laid fibre web increases from 49.1 ± 2.3 to 61.2 ± 2.5 after hydroentanglement (figure 6*f*), which is higher than that of bathroom tissue (51.2 ± 2.5). These results show that the hydroentanglement technique can improve the softness properties of wet-laid fibre web, and wet-laid hydroentangled nonwovens could be a possible alternative to bathroom tissue.

### 3.5. Air permeability, water absorption property and pore size distribution

**Figure 7.**
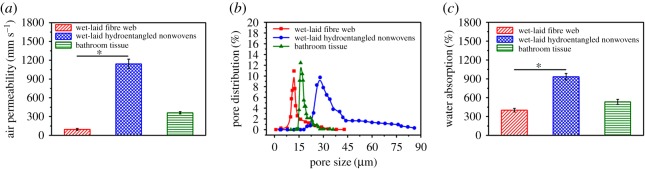
(*a*) Air permeability. (*b*) Pore size distribution. (*c*) Water absorption.

The average air permeability of the wet-laid fibre web is 95.9 ± 15.2 mm s^−1^ (figure 7*a*), which is lower that of wet-laid hydroentangled nonwovens (1140.5 ± 74.7 mm s^−1^). The air permeability shows a good agreement with the pore size results. Specifically, the pore size and pore size distribution curve of the wet-laid fibre web has a smaller well-developed peak centred at 12 µm (figure 7*b*) than that of wet-laid hydroentangled nonwovens (28 µm). This could be attributed to the process of hydroentanglement which induces the fluffy structures. In addition, the air permeability of wet-laid hydroentangled nonwovens is much higher than that of bathroom tissue (359.8 ± 19.9 mm s^−1^).

Additionally, water absorption property of bathroom tissue is compared with those of the wet-laid fibre web and wet-laid hydroentangled nonwovens (figure 7*c*), the water absorption value of bathroom tissue is 533.9% ± 39.7%, which is higher than that of the wet-laid fibre web (399.3% ± 29.8%). However, the water absorption of wet-laid hydroentangled nonwovens is the highest (932.3% ± 51.2%). This is probably owing to the formation of highly porous and fluffy structures that can hold more water for water absorption. Therefore, the wet-laid hydroentangled nonwovens show attractive properties for use as personal hygiene care materials.

### 3.6. Dusting off

**Figure 8.**
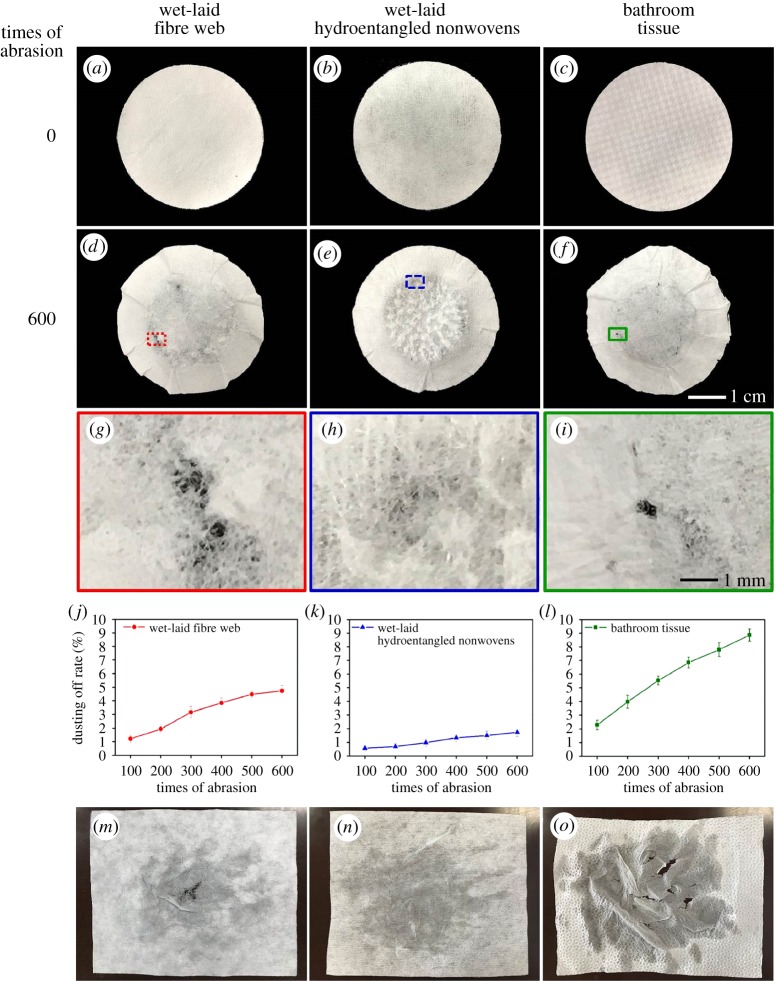
Dusting off properties of wet-laid fibre web, wet-laid hydroentangled nonwovens and bathroom tissue. (*a–c*) Samples before abrasion, (*d–f*) 600 times of abrasion, (*g–i*) higher magnification after 600 times of abrasion, (*j–l*) dusting off rate, (*m–o*) samples after wiping wet hands.

The dusting off property has been determined by an abrasion test method. Figure 8 shows different times of abrasion and dusting off rate of wet-laid fibre web, wet-laid hydroentangled nonwovens and bathroom tissue. It can be seen that these three types of materials show different appearances after 600 times of abrasion (figure 8*a–f*). Bathroom tissue and wet-laid fibre web have been abraded with small holes, while nonwovens are intact without any holes (figure 8*g–i*). The dusting off rates of wet-laid fibre web, wet-laid hydroentangled nonwovens and bathroom tissue are 4.7% ± 0.4%, 1.7% ± 0.3%, and 8.9% ± 0.5%, respectively (figure 8*j–l*). In particular, the dusting off rate of wet-laid hydroentangled nonwovens is lowest among these three materials as the fibre entangling structures can prevent the short fibres from dropping off during the abrasion process. These results prove that hydroentanglement technique can effectively decrease the dusting off rate of materials produced by the wet-laid process. Figure 8*m–o* show the application of wiping wet hands using these three materials, the wiping results are in accordance with the properties tested by abrasion experiments. Hence, wet-laid hydroentangled nonwovens as wiping materials are the best among these three materials.

### 3.7. Dispersibility

**Figure 9.**
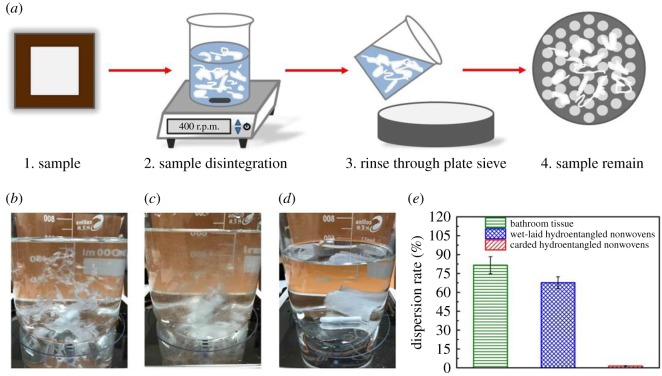
Disintegration of bathroom tissue, wet-laid hydroentangled nonwovens and carded hydroentangled nonwovens. (*a*) Schematic showing the disintegration steps. (*b*) Bathroom tissue in the beaker. (*c*) Wet-laid hydroentangled nonwovens in the beaker. (*d*) Carded hydroentangled nonwovens in the beaker. (*e*) Dispersion rate.

We next investigated the dispersibility of wet-laid hydroentangled nonwovens. For comparison, carded hydroentangled nonwovens and bathroom tissue are also tested (figure 9*a*). The disintegration of three samples in the beaker after stirring is shown in figure 9*b–d*. The dispersion rate of wet-laid hydroentangled nonwovens (67.6% ± 4.7%) is much higher than that of carded hydroentangled nonwovens (1.4% ± 0.3%, figure 9*e*). Importantly, disintegration property of wet-laid hydroentangled nonwovens is comparable to that of bathroom tissue (81.5% ± 6.8%), thus implying their potential application for hygiene personal products which can be disposed *via* the wastewater system without causing a clogging problem.

## 4. Conclusion

In summary, the pulp/Tencel wet-laid hydroentangled nonwovens with high water absorbency and good flushability for personal hygiene care are successfully fabricated via a hydroentanglement technique. The effect of hydroentanglement on the properties of wet-laid fibre web is investigated. It should be noted that wet-laid fibre web does not have obvious entanglement structures between fibres owing to the combined principles of filtration and thickening processes during the wet-laid process. After hydroentanglement, the entanglements between fibres increase and the structures of nonwovens become fluffier and tridimensional. In particular, the mechanical properties of wet-laid fibre web are greatly improved through hydroentanglement. Importantly, the softness, air permeability and water absorbency of the wet-laid fibre web are also significantly improved after hydroentanglement. The dusting off rate of wet-laid hydroentangled nonwovens is significantly reduced after consolidation and much less than that of bathroom tissue. The actual use of these materials to wipe wet hands can also prove that wet-laid hydroentangled nonwovens have good wet strength and abrasion resistance. Moreover, wet-laid hydroentangled nonwovens show good dispersibility compared with carded hydroentangled nonwovens, which is environmentally friendly to the wastewater system. Therefore, wet-laid hydroentangled nonwovens can be used as cleaning materials for personal hygiene care applications.

## Supplementary Material

The original datasets of the manuscript

## Data accessibility

The datasets supporting this article have been uploaded as part of the electronic supplementary material.
